# Married—Phares—Barfield

**Published:** 1868

**Authors:** 


### MARRIED.

Phares—Barfield.—March 5th, at the residence of the bride's father, near Clinton, La., by Rev. W. Ballard, J. H. Phares, M. D., of Newtonia, Miss,, and Miss Emily E. Barfield.

